# Deep‐Subwavelength Slot‐Enhanced Broadband Dynamic Camouflage Metasurface Across the S, C, X, and Ku Bands

**DOI:** 10.1002/nap2.70026

**Published:** 2026-02-13

**Authors:** Qiaobai He, Ruicong Zhang, Zicheng Song, Zhaoxu Pan, Zeqin Li, Yurong He, Tianyu Wang, Jiaqi Zhu

**Affiliations:** ^1^ Center for Composite Materials and Structures Harbin Institute of Technology Harbin China; ^2^ Zhengzhou Research Institute Harbin Institute of Technology Zhengzhou China; ^3^ School of Energy Science & Engineering Harbin Institute of Technology Harbin China

**Keywords:** deep‐subwavelength slot, dynamic modulation, equivalent interface‐impedance, metasurface

## Abstract

The rapid progress of microwave imaging technology has made conventional camouflage materials with fixed absorption performance ineffective. As the imaging band expands, camouflage materials capable of broadband operation, especially in the S and the C band, dynamic modulation are required to hide targets in complex environments. Here, we propose a dynamically modulated camouflage metasurface employing deep‐subwavelength slots to enhance multiband modulation capability. By varying the vertical displacement of the structure, reflectivity can be modulated from below −10 dB to near 0 dB over 2.7–19.1 GHz (150.4% relative bandwidth), while maintaining insensitivity to the incidence angle and polarization. An equivalent interface‐impedance model is established to reveal the mechanism of slot‐enhanced low‐frequency resonance. The laser processing parameters are optimized to reduce the slot width to 25 μm (*λ*_max_/4440), enabling broadband dynamic modulation, as experimentally verified. Benefiting from its broadband dynamic modulation performance extending to the S and the C band, the proposed camouflage metasurface demonstrates potential for countering emerging microwave imaging technologies.

## Graphical abstract

A deep‐subwavelength slot‐enhanced camouflage metasurface enabled by vertical displacement is demonstrated for broadband dynamic electromagnetic modulation. By introducing deep‐subwavelength slots, the operating bandwidth is significantly extended toward lower frequencies, while vertical uniaxial displacement allows continuous reflectivity modulation from −10 dB to near 0 dB across the S, C, X, and Ku bands.

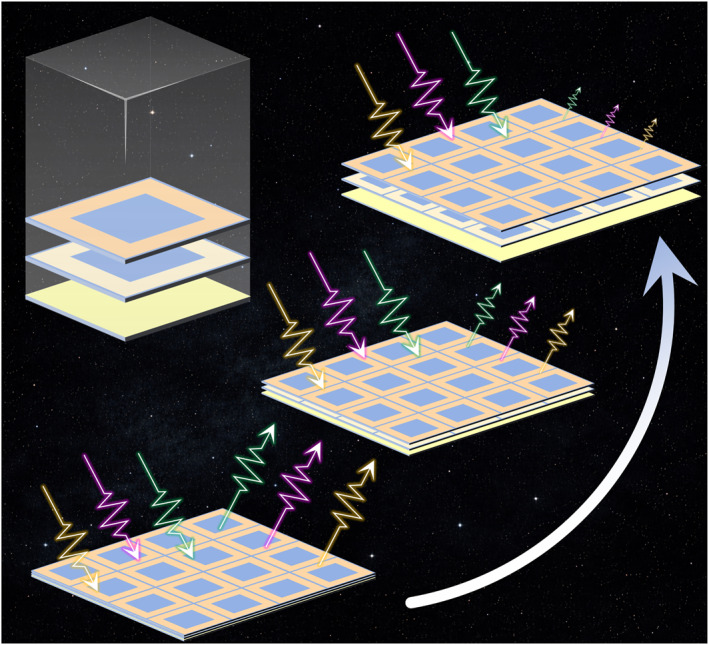

## 1 Introduction

Synthetic aperture radar (SAR) acquires high‐resolution two‐dimensional images of targets against backgrounds by integrating both the amplitude and the phase of the backscattered signals, where the image contrast is determined by the difference in reflectivity between the object and the surroundings [1, 2, 3, 4]. Conventional camouflage strategies rely on electromagnetic absorbers and scatterers with fixed reflectivity, typically ranging from −10 to −20 dB [5, 6, 7, 8, 9]. In principle, RCS (radar cross section) reduction or cloak is achieved through ohmic loss and the Pancharatnam–Berry phase, respectively [10, 11, 12, 13]. However, the electromagnetic reflectivity of natural environments varies drastically with climate and terrain. For instance, in the X band, the typical reflectivity of water surfaces, deserts, and grasslands is approximately −2 dB, −10 dB, and −18 dB, respectively [14]. Such large fluctuations make it difficult for traditional camouflage materials to remain adaptive, often resulting in the loss of invisibility under SAR imaging. Moreover, with the continuous development of SAR technology, the detection frequency band has been progressively extended to the S, C, X, and Ku bands [15, 16, 17, 18, 19]. These challenges highlight the urgent need for camouflage materials capable of broadband operation, especially in the range from 2 to 8 GHz, deep modulation, which provide a promising solution to stealth difficulties in SAR imaging.

Changing electromagnetic constants by physical fields or controlling lumped elements with external voltages makes it possible to dynamically modulate reflectivity, and significant progress has been made in recent years [13, 20, 21, 22, 23, 24, 25]. Advanced two‐dimensional materials such as graphene and vanadium dioxide (VO_2_) possess surface resistances that can be modified under external physical stimuli, thereby enabling dynamic electromagnetic modulation [26, 27, 28, 29]. For example, Kocabas used graphene to fabricate absorbing materials that realized deep reflectivity modulation from −10 dB to −60 dB under a bias voltage less than 5 V [30]. Tan patterned VO_2_ into metasurfaces, which achieved large‐range dynamic reflectivity modulation from −5 dB to −30 dB at 15 GHz by varying the temperature [31]. However, due to the narrow frequency range of the variable electromagnetic constants, dynamically modulated absorbers based on these materials are difficult to realize broadband electromagnetic modulation. Lumped elements provide another method of modulating electromagnetic waves [32, 33, 34, 35]. By applying a bias voltage to lumped elements, the surface current distribution of the camouflage materials can be changed, thereby enabling control of reflectivity. For example, Cui placed PIN diodes between strip‐shaped metal films and achieved a multifunctional reconfigurable metasurface with absorption and reflection in the 4–8 GHz band [36]. However, placing lumped elements on simple patterns can only achieve frequency tunability or narrowband amplitude modulation. Although some advanced works have realized broadband and deep modulation, they simultaneously lead to more complicated design and fabrication as well as higher cost [37, 38].

The strategy of structural deformation offers a promising route to overcome the above limitations and has attracted extensive attention from researchers [39, 40, 41, 42, 43]. Kirigami and origami metamaterials, which integrate classical folding geometries with electromagnetic metasurfaces, have been proposed to achieve dynamic manipulation of electromagnetic waves. For example, Xu and Song, respectively, fabricated kirigami‐ and origami‐inspired structures using ITO (indium tin oxide) films, both realizing broadband and large‐range reflection modulation, although their structural designs remain relatively complicated [44, 45]. Compared with such topological fold structure, uniaxial mechanical motion is simpler and easier to control. Chen innovatively proposed a planar complementary structure, where the reflectivity could be modulated from −2 dB to −18 dB by adjusting the vertical displacement between complementary layers [14]. This approach successfully emulated the X band reflectivity characteristics of diverse natural environments, representing a promising starting point for deep modulation based on uniaxial mechanical motion. Nevertheless, since the S and C bands are widely employed in practical SAR imaging, the challenge of achieving large‐range dynamic modulation at these lower frequency bands remains unresolved.

In this work, we propose a dynamically modulated camouflage metasurface based on deep‐subwavelength slots, which enables large‐range reflectivity control of electromagnetic waves across the S, C, X, and Ku bands through vertical uniaxial mechanical motion. An equivalent interface‐impedance model is developed to quantitatively describe the dynamic electromagnetic behavior and to elucidate the physical mechanism by which deep‐subwavelength slots enhance the absorption of multi‐band electromagnetic waves. In the fully expanded state, corresponding to the maximum vertical displacement, the proposed metasurface suppresses reflectivity below 10% (*S*_*11*_ < −10 dB) over a wide frequency range of 2.7–19.1 GHz. In contrast, in the fully compressed state with zero displacement, nearly complete reflection is achieved (*S*_*11*_ ≈ 0 dB). By rationally controlling the vertical expansion and contraction of the structure, the absorption ratio can be dynamically modulated from 0% to 90%. The reported broadband dynamically modulated camouflage metasurface thus addressing the long‐standing challenge of simultaneous broadband and large‐range electromagnetic modulation. Moreover, the design concept of employing deep‐subwavelength slots provides a new paradigm for improving the performance of camouflage materials in multiband.

## 2 Design and Simulation

### 2.1 Structure and Performance

**FIGURE 1:**
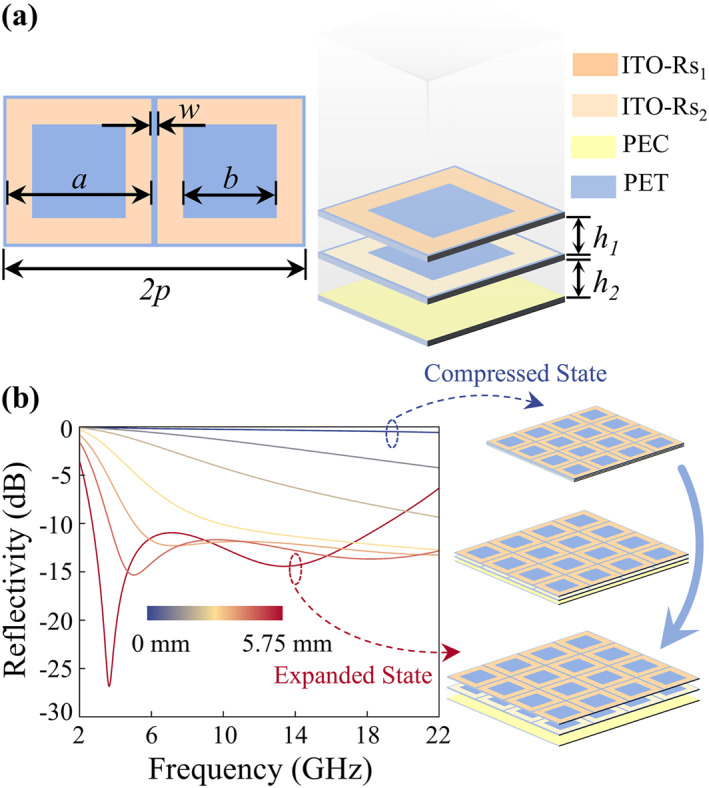
Dynamically modulated camouflage metasurface. (a) Schematic of the unit cell. (b) Structural schematics and reflection spectra in the expanded, compressed, and intermediate states.

ITO film, as a widely used transparent conductive material in various electromagnetic devices, offers high optical transparency, excellent uniformity, and low‐cost large‐scale producibility. Therefore, it is employed in the design of the proposed dynamically modulated camouflage metasurface. The metasurface is constructed as a periodical structure with deep‐subwavelength slots. The geometry of the periodic unit cell is illustrated in Figure 1a. Each unit consists of two ITO films with high sheet resistance (top and middle layers) patterned with slot arrays, together with one ITO film with low sheet resistance that acts as the reflector. The unit cell has a period of *p* = 4 mm. On both the top and middle layers, square‐ring ITO patches with identical geometrical parameters are patterned, with the outer contour side length of each square ring set to *a* = 3.975 mm. Adjacent unit cells are separated by slots of width *w* = 0.025 mm, as shown on the left of Figure 1a. The parameters *p*, *a*, and *w* satisfy the relation *p = a* *+* *w*. The inner contour side length of the square ring is *b* = 2 mm. In the fully expanded state, the separation between the top and middle ITO films is *h*_*1*_ = 5.75 mm and that between the middle ITO film and the reflector is *h*_*2*_ = 5.75 mm. All ITO films are deposited on PET (polyethylene terephthalate) substrates of thickness *d*_*PET*_ = 0.125 mm. The sheet resistance of the top ITO film is *R*_*s1*_ = 200 Ω/sq, that of the middle ITO film is *R*_*s2*_ = 100 Ω/sq, and that of the reflector is *R*_*s3*_ = 5 Ω/sq. The PET substrate has a relative permittivity of *ε*_*r*_ = 3.2 and a loss tangent of tan*δ* = 0.002 [46].

The reflection performance of the dynamically modulated camouflage metasurface under normal incidence is shown in Figure 1b. The reflectance (*R*), transmittance (*T*), and absorptance (*A*) of electromagnetic waves satisfy an energy conservation relation, *R + T + A* = 100%. Because the bottom ITO layer is approximated as a PEC (perfect electric conductor), the transmittance of electromagnetic waves is approximately zero, and the relation reduces to *A* + *R* = 100%. In the fully expanded state (*h*_*1*_ = *h*_*2*_ = 5.75 mm), the metasurface reflects less than 10% of the incident waves within 2.7–19.1 GHz (*A* > 90%). In the fully compressed state (*h*_*1*_ = *h*_*2*_ = 0), nearly complete reflection (*A* ≈ 0) is achieved in the same frequency band. To verify that electromagnetic absorption of the structure can be continuously modulated by adjusting the vertical displacement of the films, the absorption spectra under normal incidence are calculated for the case *h*_*1*_ = *h*_*2*_, as illustrated in Figure 1b. As the displacement decreases from 5.75 to 0 mm, the reflectivity across 2.7–19.1 GHz changes continuously from below −10 dB to near 0 dB, corresponding to a gradual reduction of absorption from above 90% to nearly vanishing.

### 2.2 Model and Analysis

**FIGURE 2:**
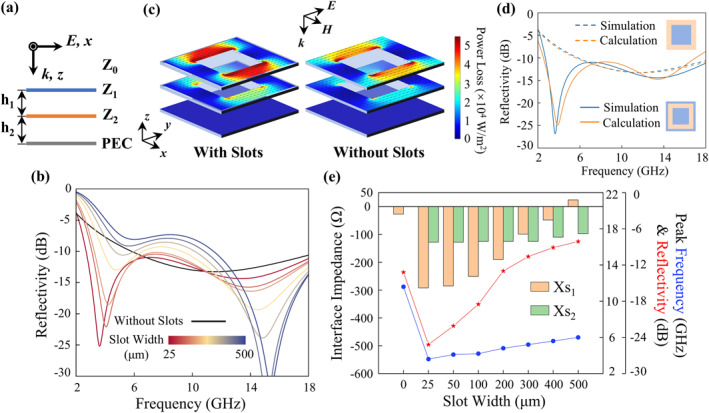
The equivalent interface‐impedance model and physical explanation. (a) Equivalent interface ‐impedance model. (b) Comparison of reflection spectra for metasurfaces without slots and with different slot widths. (c) Surface power loss density and current density distributions at the 3.61 GHz for the structure with 25 μm slots (left) and without slots (right). (d) Comparison of reflection results obtained from numerical simulation and model calculation. (e) Variations of the imaginary part of the interface impedance, absorption peak, and absorption strength with slot width.

To explain the physical mechanism by which the vertical displacement dynamically modulates the electromagnetic response and the slots enhance the low‐frequency absorption, an equivalent interface‐impedance model is proposed. The structure can be represented by a physical model of “PEC–air–impedance boundary 2–air–impedance boundary 1.” As shown in Figure 2a, in this model, the impedance of boundary 1 is given by *Z*_*1*_ *= R*_*s1*_ *+* *iX*_*s1*_, the impedance of boundary 2 by *Z*_*2*_ *= R*_*s2*_ *+* *iX*_*s2*_, and the impedance of air by *Z*_*0*_. When a plane wave $\vec{E}=E_{0}e^{-ik_{0}z}\hat{x}$ is normally incident along the *z*‐axis from the air outside boundary 1, a reflected wave ${\vec{E}}_{r}=E_{r}e^{ik_{0}z}\hat{x}$ is simultaneously generated. The reflectivity *R* is defined as ${\left|\frac{E_{r}}{E_{0}}\right|}^{2}$. It can be derived from the Maxwell boundary conditions of each layer. For ease of comparison with the simulation results, the reflectivity is expressed in decibels; *R*_*dB*_ is used, which is essentially equivalent to *S*_*11*_. The derivation process is shown in Section S1 (Supporting Information S1), and *R*_*dB*_ is expressed by Equation (1):

(1) $$R_{dB}=10\;\mathrm{lg}\left({\left|\frac{\left(2-\gamma \right)\left[\left(2-\xi \right)+\xi e^{2ik_{0}h_{2}}\right]-\gamma e^{2ik_{0}h_{1}}\left[\xi -\left(2+\xi \right)e^{2ik_{0}h_{2}}\right]}{\left(2+\gamma \right)e^{2ik_{0}h_{1}}\left[\xi -\left(2+\xi \right)e^{2ik_{0}h_{2}}\right]+\gamma \left[\left(2-\xi \right)+\xi e^{2ik_{0}h_{2}}\right]}\right|}^{2}\right),$$

where *γ* = *Z*_*0*_ */Z*_*1*_ denotes the ratio of the air impedance to the impedance of boundary 1, and *ξ* = *Z*_*0*_ */Z*_*2*_ denotes the ratio of the air impedance to the impedance of boundary 2. The model is employed to analyze how the vertical displacement of the films alters the absorption–reflection characteristics of the metasurface. When the displacement is nonzero (*h*_*1*_ ≠ 0 and *h*_*2*_ ≠ 0), *R*_*dB*_ < 0, indicating absorption of the incident electromagnetic waves; when there is no vertical displacement (*h*_*1*_ = *h*_*2*_ = 0), $\lim_{h_{1},h_{2}\to 0}R_{dB}=0$, and the structure completely reflects electromagnetic waves at all frequencies.

The slot width is the main factor affecting the metasurface performance, exerting a significant influence on both the position and the depth of the low‐frequency absorption peak. As shown in Figure 2b, the reflection curves were calculated for metasurfaces without slots and with slot widths *w* varying from 25 to 500 μm. The metasurface without slots exhibits absorption greater than 90% only around 12 GHz, whereas the metasurface with a 25‐μm slot extends the operating bandwidth down to 2.7 GHz and produces an absorption peak exceeding 99% at 3.61 GHz. As the slot width increases, the low‐frequency absorption peak shifts toward higher frequencies whereas the absorptivity decreases rapidly. As shown in Figure 2c, the surface power loss density distributions under normal incidence of TE‐polarized (*E*_*y*_) waves at the peak of 3.61 GHz were calculated and compared before and after introducing the slots. It can be seen that the slots excite strong electromagnetic resonances on the two lossy films at 3.61 GHz, enhancing the electric field intensity and thereby improving the low‐frequency absorption performance of the metasurface, with the top film providing the main contribution to the loss.

As shown in Figure 2d, when *Z*_*1*_ = 291–292*i* (Ω) and *Z*_*2*_ = 246–128*i* (Ω), Equation (1) fits well with the simulated reflection results of the metasurface with a slot width of 25 μm in the expanded state. When *Z*_*1*_ = 340–27*i* (Ω) and *Z*_*2*_ = 175 (Ω), Equation (1) agrees well with the simulated reflection results of the metasurface without slots. Therefore, the above model, by treating the interface impedances as constants, can quantitatively describe the influence of slot width on the reactance characteristics of the two lossy films near the low‐frequency resonance peak. The interface impedances of the lossy films with different slot widths were fitted, and Figure 2e shows the absorption peak position, the corresponding reflectivity, and the imaginary part of the interface impedances, namely the interface reactances *X*_*s1*_ and *X*_*s2*_. The negative values of *X*_*s1*_ and *X*_*s2*_ indicate that the slots introduce a phase delay of the electric field waves at the interfaces, resulting in capacitive reactance. Large capacitive reactance can induce strong low‐frequency resonance of electromagnetic waves. For the films without slots, the two interface reactances are close to zero, whereas introducing 25 μm slots abruptly decreases *X*_*s1*_ and *X*_*s2*_ to −292 (Ω) and −128 (Ω), respectively. This indicates that the low‐frequency absorption peak originates from the increase in capacitance at the interface caused by the slots. As the slot width increases, the interface capacitance gradually decreases, causing the resonance peak to shift toward higher frequencies and the absorption strength to weaken. Therefore, deep‐subwavelength slots can significantly enhance the low‐frequency absorption performance of the metasurface.

### 2.3 Oblique Incidence and Polarization

**FIGURE 3:**
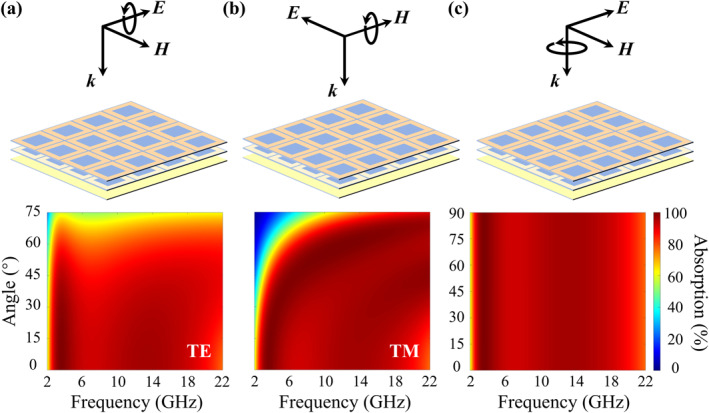
Absorption performance of the dynamically modulated camouflage metasurface under oblique incidence and at different polarization angles under normal incidence. (a) TE wave. (b) TM wave. (c) Different polarization angles.

As shown in Figure 3a,b, the absorption performance of the camouflage metasurface under oblique incidence (0°–75°) for TE and TM waves is presented. For TE wave incidence with *θ* ≤ 60°, the absorption remains above 80% across 2.7–19.1 GHz. For TM wave incidence, the absorption exceeds 90% up to *θ* = 45°; although a noticeable blue shift of the absorption band appears at 45°–60°, broadband absorption above 90% is still maintained. In Figure 3c, the absorption spectra at normal incidence versus the polarization angle are shown. Because the geometry possesses fourfold rotational symmetry, only polarizations between 0° and 90° need to be considered, and identical absorption is observed for all polarization angles.

## 3 Manufacturing and Characterization

**FIGURE 4:**
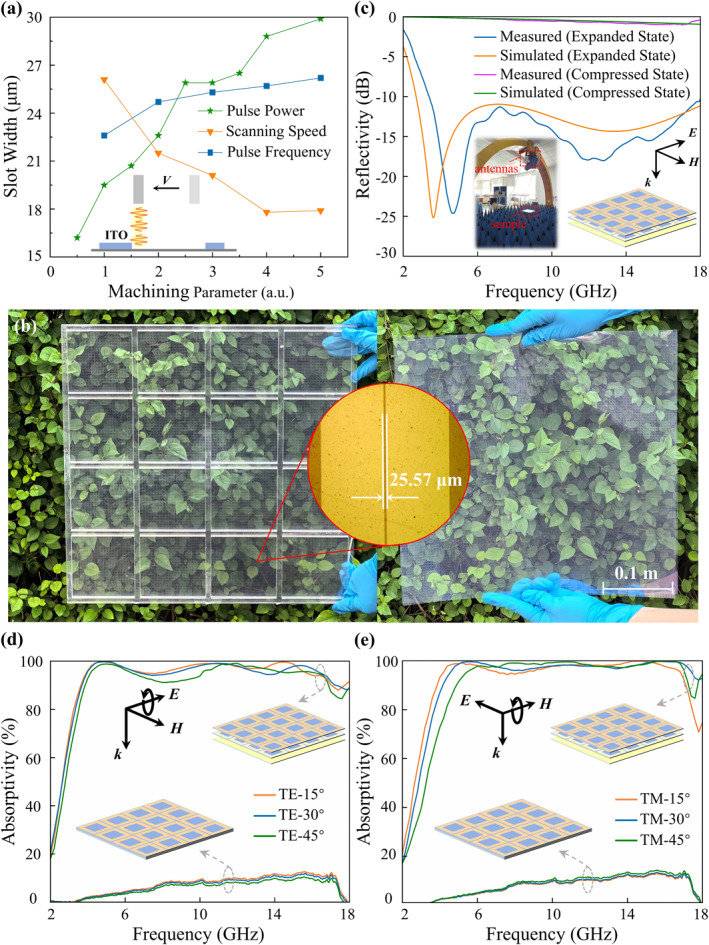
Fabrication and testing of the dynamically modulated camouflage metasurface. (a) Dependence of slot width on the main laser‐etching parameters. The horizontal axis represents the unit values of three parameters: pulse power (5%), scanning speed (1000 mm/s), and repetition frequency (100 kHz). (b) Photographs of the samples in the expanded and compressed states. (c) Measured reflectivity spectra of the metasurface in the expanded and compressed states under normal incidence. (d) and (e) show the absorption spectra of the sample under oblique incidence for TE‐ and TM‐polarized waves, respectively.

To verify the characteristics of multiband electromagnetic absorption enhanced by slots, as well as the broadband absorption modulation induced by vertical displacement, two samples with dimensions of 400 mm × 400 mm were fabricated: one with two 5.75 mm air spacers and the other without air spacers. Each sample consisted of 100 × 100 periodic unit cells. The substrates of ITO films were all 0.125 mm thick PET. Deep‐subwavelength slots and square ring patches were patterned on the middle and top ITO layers by laser etching. An infrared nanosecond laser (operating wavelength 1064 nm) was used to scan the central square region of each unit cell, completely ablating the ITO film and exposing the underlying PET. The laser then scanned along the outer square contour at the cell boundaries, resulting in a square‐ring array with slots. It should be noted that the linewidth of the ITO etched by the infrared laser was on the same order of magnitude as the designed slot width, so the slots were represented as thin lines in the design layout. The linewidth was mainly determined by three process parameters: pulse power *P*, scanning speed *V*, and pulse repetition frequency *f*. The influence of different process parameters on the etched linewidth was investigated, and the results are shown in Figure 4a. The linewidth increases with pulse power and pulse repetition frequency, but decreases with scanning speed. As illustrated in the inset of Figure 4a, during the laser etching of the ITO film, higher pulse power leads to stronger lateral heat diffusion from the center of the etched line, thereby broadening the linewidth. Conversely, a faster scanning speed or a lower pulse repetition frequency reduces the number of laser pulses delivered per unit length, resulting in narrower etched lines. These observations qualitatively explain the experimental trends of how the process parameters affect the linewidth. Based on these results, the parameters of 25% pulse power, 1000 mm/s scanning speed, and 200 kHz repetition frequency were selected to etch slots in the middle and top ITO layers. Figure 4b shows the fabricated camouflage metasurface samples in the expanded state (left) and the compressed state (right). To emulate the expanded state, two 5.75 mm‐thick PMMA (poly(methyl) methacrylate) frames were inserted between the three ITO layers; when the PMMA frames were removed, the three ITO films were stacked directly to emulate the compressed state. Section S2 (Supporting Information S1) provides a design scheme for a mechanical structure used to control the vertical displacement of the ITO films. The inset presents an optical microscope image of the slots, where parallel‐line measurement yields an average slot width of 25.57 μm, consistent with the designed value. Through the samples, the background bushes can be clearly seen.

The arch method was employed to measure the reflection spectra of the sample in the expanded and compressed states under normal incidence. As shown in the inset of Figure 4c, a linearly polarized horn antenna mounted on the arch and connected to a vector network analyzer was used to measure the reflectivity of the samples. An aluminum plate of the same size was used for normalization. As plotted in Figure 4c, the experimental results indicate that within the operating band, the sample in the expanded state exhibits a reflectivity below 10% under normal incidence (*A* > 90%), whereas the sample in the compressed state reflects nearly all the incident electromagnetic energy. The simulated results are in good agreement with the experimental measurements. Figure 4d and e present the measured reflectivity spectra of the sample under oblique incidence for TE‐ and TM‐polarized waves, respectively. The experimental results show that in the expanded state, the sample maintains an absorption higher than 90% for TE‐polarized waves with incident angles up to 45° and also achieves absorption exceeding 90% for TM‐polarized waves within 45° incidence, although a blue shift of the absorption band is observed. The measured results exhibit the same trends as the simulation in Section 2.3. In addition, the reflection performance of the sample in the compressed state is insensitive to both polarization and incident angles, which can be attributed to the three ITO films being compressed together. In summary, the experiments confirm that by controlling the vertical displacement of the structure, the reflection can be modulated from below −10 dB to near 0 dB, thereby realizing continuous absorption–reflection modulation.

**FIGURE 5:**
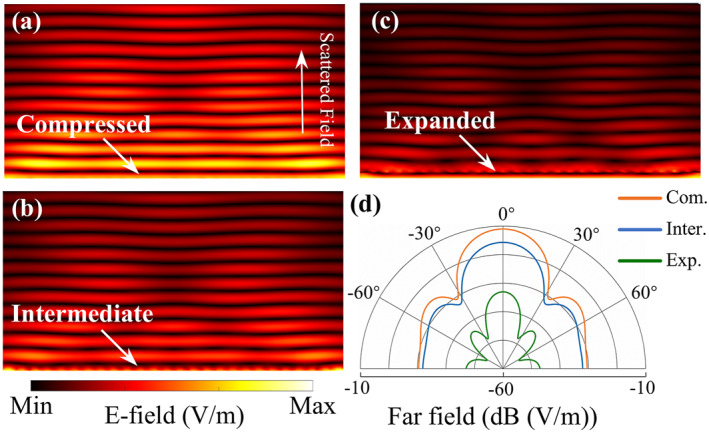
Dynamic camouflage performance of the metasurface. (a–c) Near‐field scattering distributions of the metasurface in the compressed, intermediate (*h*_*1*_ = *h*_*2*_ = 1 mm), and expanded states under a normally incident plane wave at 12.5 GHz. (d) Corresponding far‐field scattering patterns.

To verify the dynamic camouflage performance of the metasurface, the calculated near‐field and far‐field scattering patterns of the metasurface in the compressed, intermediate, and expanded states under normally incident plane waves are presented in Figure 5. As shown in Figure 5a–c, with increasing vertical displacement of the ITO films, the scattered field in the near‐field region is progressively suppressed owing to enhanced absorption of the incident electromagnetic waves. Correspondingly, Figure 5d demonstrate that the suppression level of the far‐field scattering in all directions can be continuously modulated. Therefore, the proposed metasurface exhibits excellent dynamic control over the scattering field. In complex environments, the dynamic camouflage metasurface can achieve adaptive modulation of the scattering field by controlling the vertical displacement of the ITO films, indicating strong potential for broadband dynamic camouflage against microwave imaging.

To demonstrate the improvements of the proposed camouflage metasurface, several recent works on mainstream electromagnetic modulation approaches are summarized in Table 1. The table compares this work with five representative studies in terms of operating frequency band, modulation depth, relative thickness with respect to the Rozanov limit (*d*_*Roz*_) [49], and optical transparency. The proposed dynamically modulated camouflage metasurface not only offers a larger bandwidth and modulation depth, but also combines the advantages of low relative profile, optical transparency, simple design, and easy fabrication, thereby providing a practical solution for stealth under SAR imaging.

**TABLE 1:** Comparison of different approaches to dynamic modulation.

Ref.	Approach	Material	Operation frequency	Modulation depth	Thickness and Thickness/*d*_*Roz*_	Transparency	Year
[47]	Temperature	VO_2_	Narrowband	50 dB	2.5 mm and N.A.	Yes	2024
[48]	Voltage	Graphene	9.9–27.7 GHz (95%)	Over 10 dB	4 mm and 2.24	No	2024
[36]	Circuit	PIN diode	4–8 GHz (67%)	Over 10 dB	12 mm and 2.72	No	2025
[37]	Circuit	PIN diode	1.5–6.5 GHz (125%)	Over 7 dB	44.5 mm and 3.78	No	2025
[14]	Mechanics	ITO	8–12 GHz (40%)	Over 15 dB	7 mm and < 3.17	Yes	2024
Ours	Mechanics	ITO	2.7–19.1 GHz (150%)	Over 10 dB	11.5 mm and 1.76	Yes	2025

## 4 Conclusion

In this work, a dynamically modulated camouflage metasurface was designed by introducing deep‐subwavelength slots to broaden the electromagnetic modulation bandwidth. The metasurface allows the reflection (or absorption) to be modulated by controlling the vertical displacement between two ITO films. Within the frequency range of 2.7–19.1 GHz, continuous absorption modulation from near 0% to above 90% (*S*_*11*_ ≈ 0 dB to −10 dB) was achieved. In the expanded state, the metasurface maintains broadband absorption for TE and TM waves incident up to 60° within the operating band. Furthermore, an equivalent interface‐impedance model was proposed to elucidate the physical mechanism of slot‐enhanced low‐frequency absorption. The deep‐subwavelength slots significantly increase the capacitive reactance at the interface, leading to strong low‐frequency resonances that boost absorption. As the slot width increases, the capacitance decreases, resulting in a blue shift and weakened resonance strength of the absorption peak. In the fabrication study, the laser‐etching parameters for producing micron‐scale slots were investigated, and the effects of pulse power, scanning speed, and repetition frequency on slot width were systematically characterized. The proposed dynamically modulated camouflage metasurface features large‐range reflection modulation and a simple design and fabrication process, providing an effective solution for camouflage materials under broadband microwave imaging.

## Supporting information


Supporting Information S1

## Data Availability

The data that support the findings of this study are available from the corresponding author upon reasonable request.
