# Supplementary material for: Deep‐Subwavelength Slot‐Enhanced Broadband Dynamic Camouflage Metasurface Across the S, C, X, and Ku Bands
Source: Nanophotonics. 2026 Feb 13;15(4):e70026. doi: 10.1002/nap2.70026 (PMC12964986; doi:10.1002/nap2.70026)
Supplement: Supplementary file 1 — Supporting Information S1 [file NAP2-15-e70026-s001.docx]

Supporting Information

Deep-subwavelength slot-enhanced dynamic camouflage metasurface enabled by vertical displacement for broadband modulation across the S, C, X, and Ku bands

Qiaobai He, Ruicong Zhang, Zicheng Song*, Zhaoxu Pan, Zeqin Li, Yurong He, Tianyu Wang*, and Jiaqi Zhu*

**S1: Derivation of Equation 1**

The analysis is carried out based on the physical model shown in **Figure 3a**. For convenience in calculation, the *x–y* plane is set on impedance boundary 1. A plane wave $E_{i}e^{-ik_{0}z}\hat{x}$ propagating along the +z axis is incident on the structure and generates a reflected wave $E_{r}e^{ik_{0}z}\hat{x}$. The reflectivity is expressed as the ratio of the electric-field amplitude of the reflected wave to that of the incident wave:

$R_{dB}=10\lg(E_{r}/E_{i})^{2}$ (S1)

In free space and the two air spacer layers, waves propagate in the +z and −z directions. Therefore, the electromagnetic field in free space can be expressed as:

$\overrightarrow{E}_{0}=E_{i}e^{-ik_{0}z}+E_{r}e^{ik_{0}z}\hat{x}$ (S2)

$\overrightarrow{H}_{0}=H_{i}e^{-ik_{0}z}-H_{r}e^{ik_{0}z}\hat{y}$ (S3)

The electromagnetic field in the region between boundary 1 and boundary 2 (Region 1):

$\overrightarrow{E}_{1}=E_{1}^{+}e^{-ik_{0}z}+E_{1}^{-}e^{ik_{0}z}\hat{x}$ (S4)

$\overrightarrow{H}_{1}=H_{1}^{+}e^{-ik_{0}z}-H_{1}^{-}e^{ik_{0}z}\hat{y}$ (S5)

The electromagnetic field in the region between boundary 2 and the PEC (Region 2):

$\overrightarrow{E}_{2}=E_{2}^{+}e^{-ik_{0}z}+E_{2}^{-}e^{ik_{0}z}\hat{x}$ (S6)

$\overrightarrow{H}_{2}=H_{2}^{+}e^{-ik_{0}z}-H_{2}^{-}e^{ik_{0}z}\hat{y}$ (S6)

In each region, the amplitudes of the electric and magnetic fields should satisfy the following equations:

$\overrightarrow{E}=Z_{0}\overrightarrow{H}\times\hat{z}$ (S7)

Meanwhile, at every boundary, both the continuity of the tangential electric field and the impedance relation must be fulfilled:

$\hat{n}\times(\overrightarrow{E}_{A}-\overrightarrow{E}_{B})=0$ (S8)

$\hat{n}\times(\overrightarrow{H}_{A}-\overrightarrow{H}_{B})=\overrightarrow{J}_{s}=\overrightarrow{E}_{t}/Z_{s}$ (S9)

where $\hat{n}$ is the unit normal vector directed from medium A toward medium B.

By substituting the electromagnetic field expressions at $z=0$ and $z=h_{1}$ into Equation (S8) and (S9), four equations can be obtained as follows:

$E_{i}+E_{r}=E_{1}^{+}+E_{1}^{-}$ (S10)

$(H_{i}-H_{r})-(H_{1}^{+}-H_{1}^{-})=(E_{1}^{+}+E_{1}^{-})/Z_{s1}$ (S11)

$E_{1}^{+}e^{-ik_{0}h_{1}}+E_{1}^{-}e^{ik_{0}h_{1}}=E_{2}^{+}e^{-ik_{0}h_{1}}+E_{2}^{-}e^{ik_{0}h_{1}}$ (S12)

$(H_{1}^{+}e^{-ik_{0}h_{1}}-H_{1}^{-}e^{ik_{0}h_{1}})-(H_{2}^{+}e^{-ik_{0}h_{1}}-H_{2}^{-}e^{ik_{0}h_{1}})=(E_{2}^{+}e^{-ik_{0}h_{1}}+E_{2}^{-}e^{ik_{0}h_{1}})/Z_{s2}$ (S13)

In addition, it should be noted that the tangential electric field vanishes on the PEC surface, leading to the following relation:

$E_{2}^{+}e^{-ik_{0}(h_{1}+h_{2})}+E_{2}^{-}e^{ik_{0}(h_{1}+h_{2})}=0$ (S14)

By combining Equation (S10-S14), a system of five equations is formed with six unknowns $E_{i}$, $E_{r}$, $E_{1}^{+}$, $E_{1}^{-}$, $E_{2}^{+}$, and $E_{2}^{-}$. Therefore, the ratio between $E_{r}$ and $E_{i}$ can be obtained as follows:

$R_{dB}=10\lg(\left|\frac{(2-\gamma)[(2-\xi)+\xi e^{2ik_{0}h_{2}}]-\gamma e^{2ik_{0}h_{1}}[\xi-(2+\xi)e^{2ik_{0}h_{2}}]}{(2+\gamma)e^{2ik_{0}h_{1}}[\xi-(2+\xi)e^{2ik_{0}h_{2}}]+\gamma[(2-\xi)+\xi e^{2ik_{0}h_{2}}]}\right|^{2})$ (S15)

where *γ* = *Z_0_/Z_1_*, *ξ* = *Z_0_/Z_2_*.

**S2: A Kind of Mechanical Structure for Controlling the Motion of ITO Films**

As shown in **Figure S1**, the blue regions indicate the locations where the ITO films are placed. The structure is designed based on a gear–linkage mechanism, in which the gear serves as the driving component to induce eccentric motion of the linkage. This motion drives the sliders mounted on the linkage to move horizontally along their respective guide rails. Constrained by the four supporting pillars, the frame used to fix the films follows the linkage and undergoes uniaxial vertical displacement, thereby enabling dynamic electromagnetic modulation.


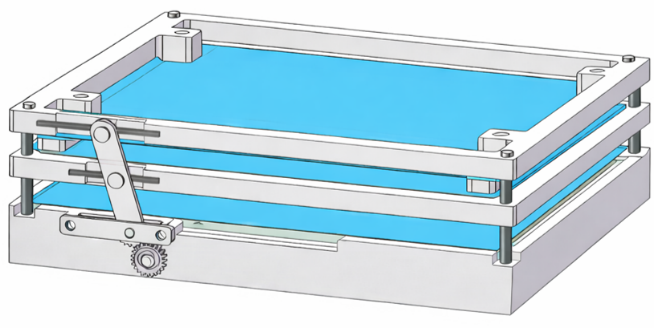


**Figure S1.** Schematic of the designed mechanical structure for controlling the vertical displacement of the three ITO films.
